# Supplementary material for: METTL14-upregulated miR-6858 triggers cell apoptosis in keratinocytes of oral lichen planus through decreasing GSDMC
Source: Commun Biol. 2023 Sep 23;6:976. doi: 10.1038/s42003-023-05360-6 (PMC10517968; doi:10.1038/s42003-023-05360-6)
Supplement: Supplementary file 2 — Supplemental Materials [file 42003_2023_5360_MOESM2_ESM.pdf]

Supplementary figures

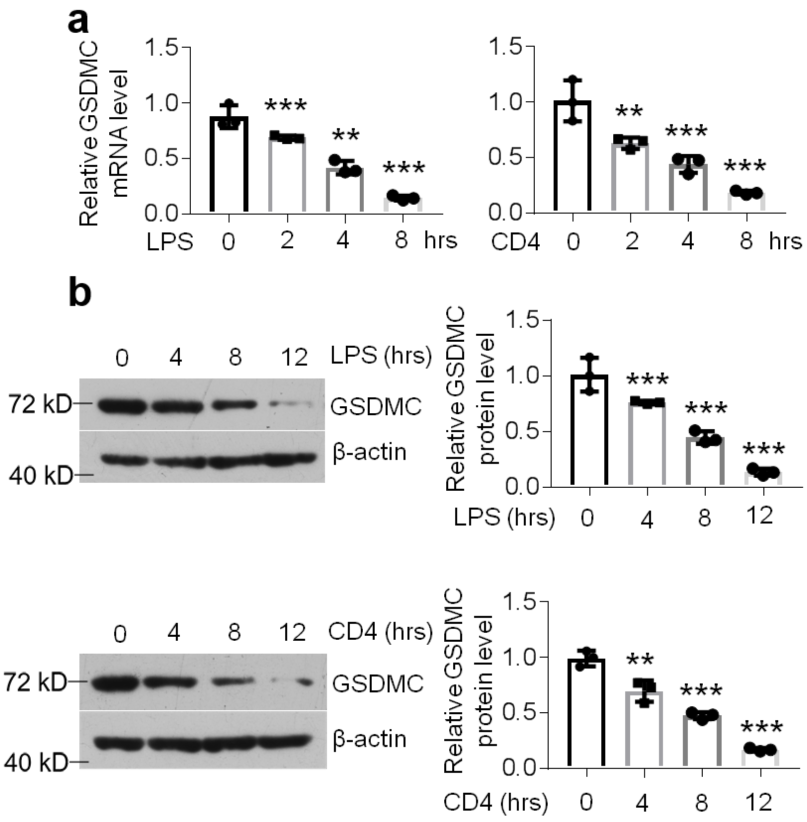

Supplementary figure 1. GSDMC expression is decreased in a time-course dependent manner in oral keratinocytes. (a-b) Real-time PCR (a) or western blot (b) measurement of GSDMC levels in HOKs treated with 100 ng/ml LPS or activated $CD4^+$ T cells at indicated time points. $^{**}P < 0.01$, $^{***}P < 0.001$ vs corresponding control group; n = 3. Data were expressed as means ± standard deviation. All experiments were carried out at least 3 times. One-way ANOVA (a and b) was used for statistical analysis.

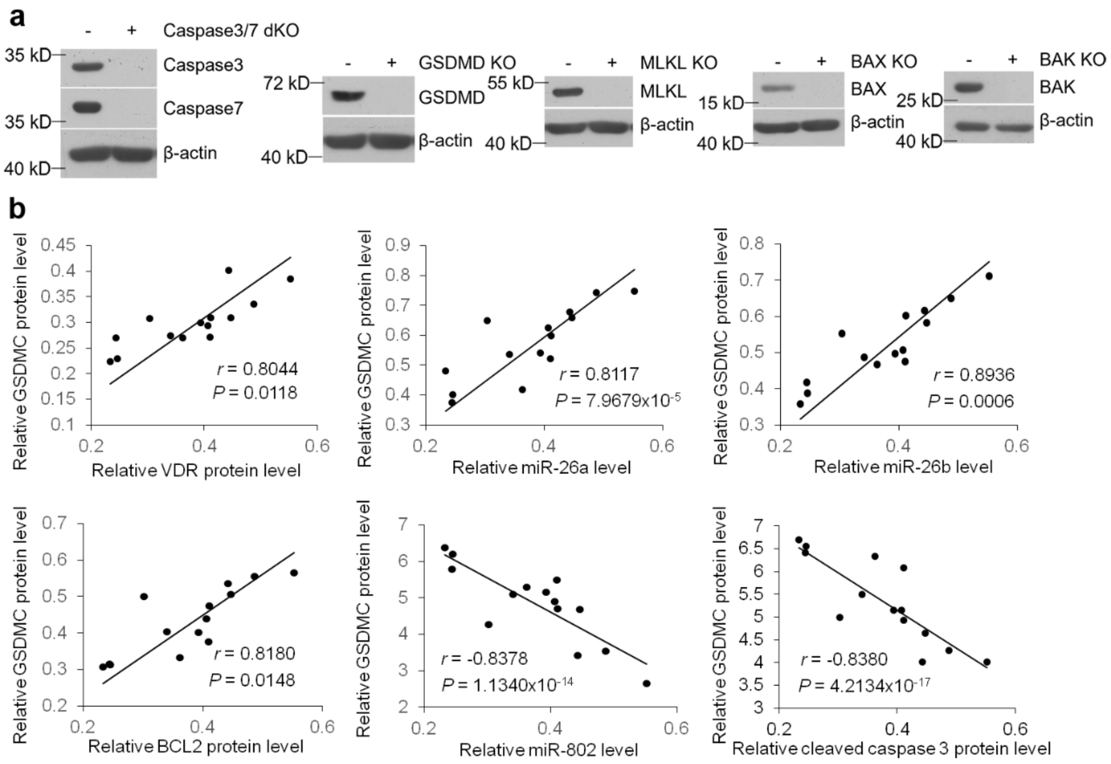

Supplementary figure 2. Knockout verification and correlations between GSDMC and other apoptosis-related factors. (a) Western blot analyses of HOKs with Caspase3/7, GSDMD, MLKL, BAX or BAK knockout. (b) Correlation analyses between GSDMC expression and VDR, miR-26a, miR-26b, BCL2, miR-802 or cleaved caspase 3 levels in the oral keratinocytes of human samples, n = 14. All experiments were carried out at least 3 times. Student's *t* test was used for statistical analysis.

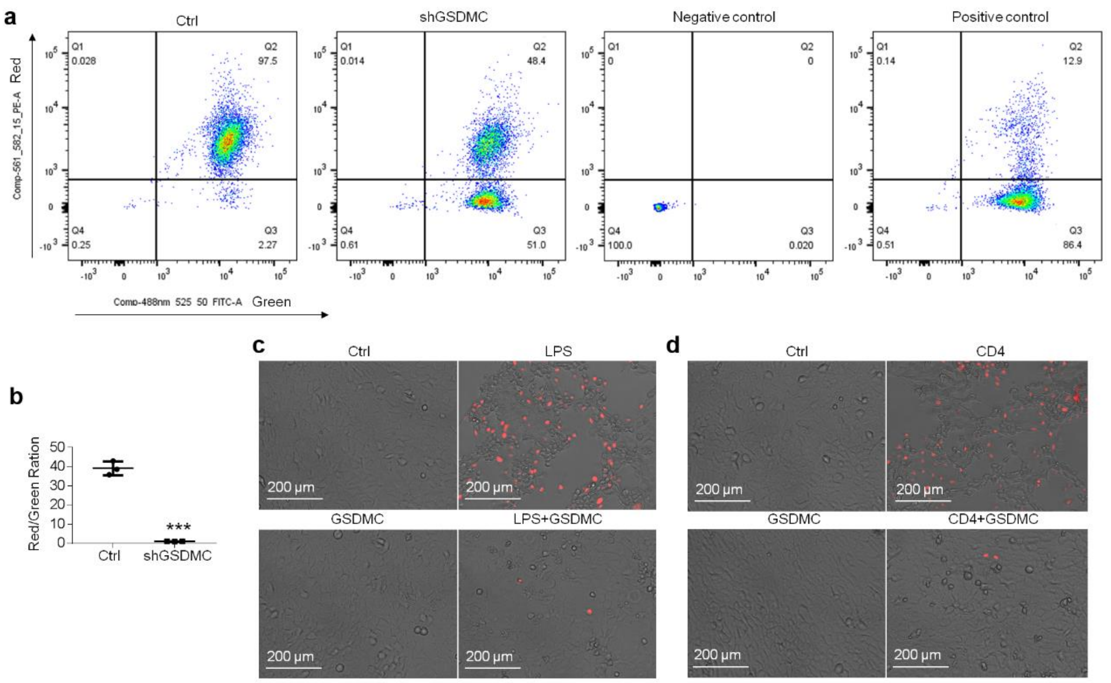

Supplementary figure 3. GSDMC has effects on mitochondrial function and cell apoptosis. (a-b) Flow cytometry analyses (a) and Red/Green ratio (b) of mitochondrial membrane potentials in HOKs with or without GSDMC knockdown. (c-d) PI staining of control- or GSDMC-lentivirus-transduced HOKs with or without 12-hour 100 ng/ml LPS (c) or CD4 T cells (d) challenge. ***$P < 0.001$ vs corresponding control group; n = 3; Ctrl, control. Data were expressed as means ± standard deviation. All experiments were carried out at least 3 times. Student's *t* test was used for statistical analysis.

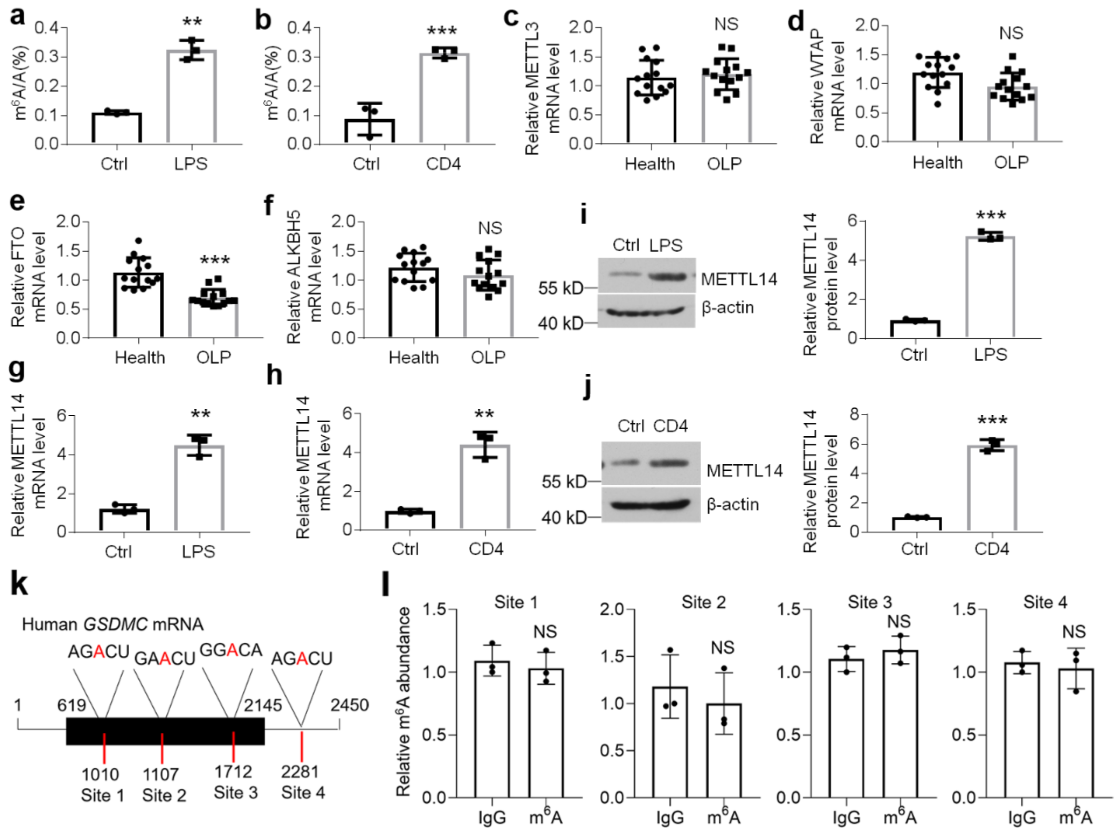

Supplementary figure 4. m^{6}A modification and METTL14 levels in oral keratinocytes. (a-b) Quantitation of m^{6}A level in total RNAs isolated from HOKs with 100 ng/ml LPS (a) or activated CD4$^+$ T cells treatment (b) for 12 hours, n = 3. (c-f) Real-time PCR analyses showing *METTL3* (c), *WTAP* (d), *FTO* (e) or *ALKBH5* (f) mRNA expression in oral keratinocytes from human mucosa, n = 14. (g-h) *METTL14* mRNA levels in HOKs with 12-hour 100 ng/ml LPS (g) or activated CD4$^+$ T cells (h) challenge determined by real-time PCR, n = 3. (i-j) METTL14 protein levels in HOKs with 12-hour 100 ng/ml LPS (i) or activated CD4$^+$ T cells (j) challenge determined by western blot and quantification analysis, n = 3. (k) Schematic showing the 4 putative m^{6}A sites in human *GSDMC* mRNA. 3 sites are at coding sequence (CDS) region and the last one is at 3'untranslated region (3'UTR). (l) m^{6}A-IP-qPCR showing m^{6}A abundance of *GSDMC* in HOKs, n = 3. $^{**}P < 0.01$, $^{***}P < 0.001$ vs corresponding

control or health group; Ctrl, control. Data were expressed as means ± standard deviation. All experiments were carried out at least 3 times. Student’s *t* test was used for statistical analysis.

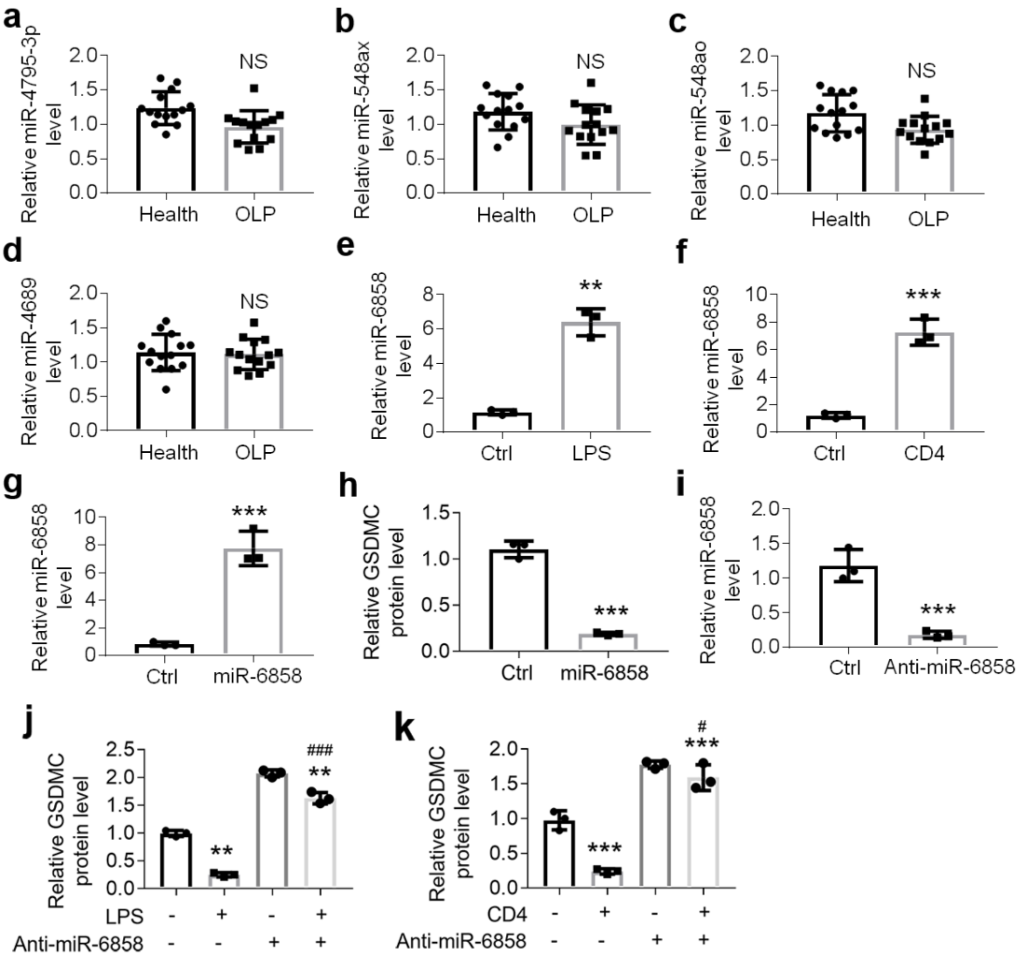

Supplementary figure 5. Expression of miRNAs in OLP. (a-d) Real-time PCR analyses exhibiting miR-4795-3p (a), miR-548ax (b), miR-548ao (c) or miR-4689 (d) levels in epithelial cells of human oral mucosa, n = 14. (e-f) miR-6858 expression in HOKs with 12-hour 100 ng/ml LPS (e) or activated $CD4^+$ T cells (f) treatment assessed by real-time PCR, n = 3. (g) miR-6858 levels in HOKs with or without 200 nM miR-6858 mimics, n = 3. (h) Quantification analysis of GSDMC levels, related to Fig. 5e, n = 3. (i) miR-6858 levels in HOKs with or without 200 nM miR-6858 inhibitors, n = 3. (j) Quantification analysis of western blot, related to Fig. 5g, n = 3. (k) Quantification analysis of western blot, related to Fig. 5i, n = 3. $^{**}P < 0.01$, $^{***}P < 0.001$ vs corresponding control or health group; $\#P < 0.05$, $\#\#\#P < 0.001$ vs

corresponding LPS or CD4 group; Ctrl, control. Data were expressed as means ± standard deviation. All experiments were carried out at least 3 times. Student's *t* test (a-i) and one-way ANOVA (j-k) were used for statistical analysis.

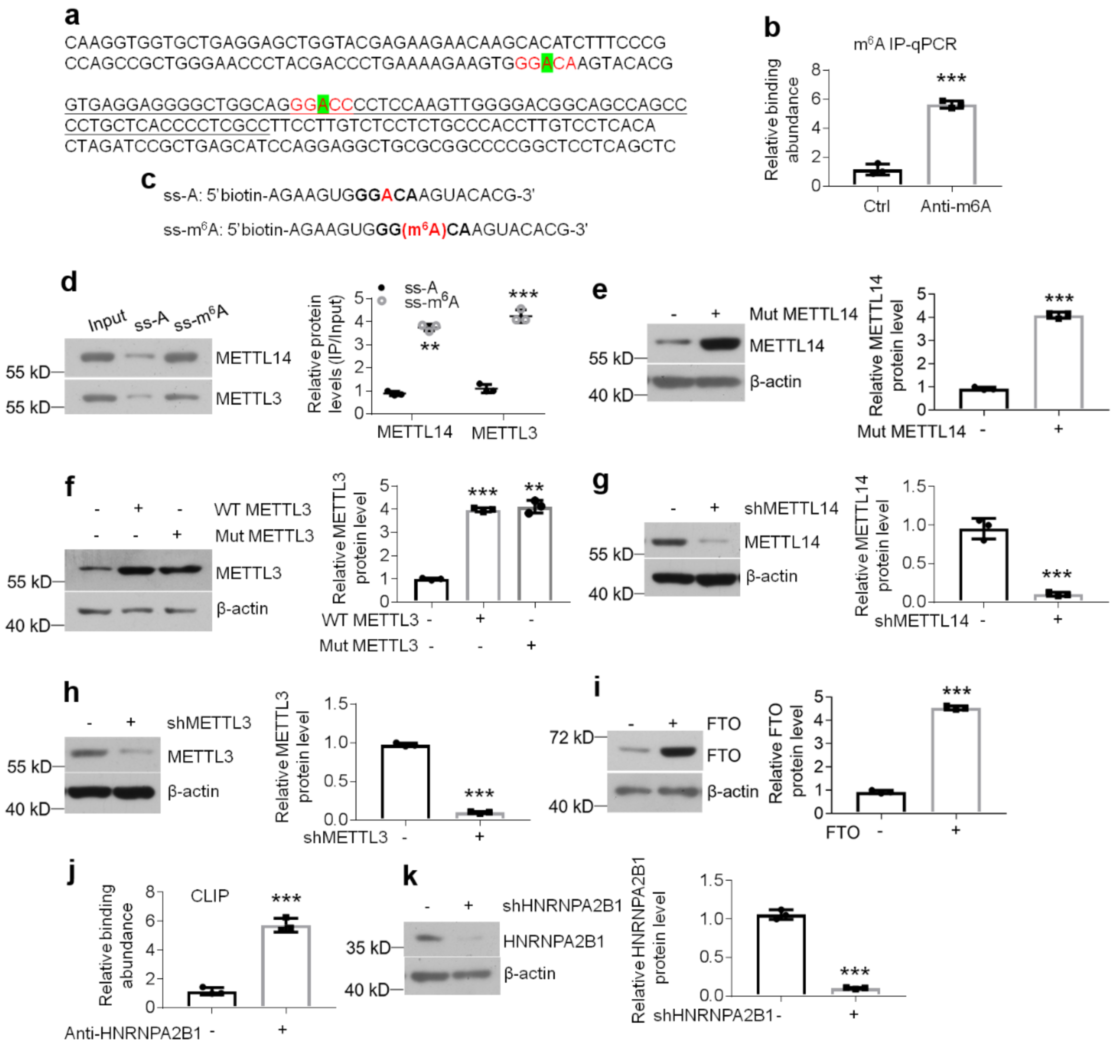

Supplementary figure 6. m^{6}A sites were located in primiR-6858. (a) Sequences of potential m^{6}A sites in primiR-6858. The m^{6}A motifs were labelled in red, *N*^{6}-Adenosine was labelled in green, premiR-6858 was underlined. (b) RIP-qPCR assays validating the predicted m^{6}A site in primiR-6858. (c) Schematic displaying ssRNA probes with unmethylated or methylated adenosine. (d) Western blotting and densitometric analysis showing the pulldown proteins expression from HOKs nuclear extract by ssRNA probes. (e-i) Western blot and densitometric analysis showing METTL3, METTL14 or FTO expression in HOKs infected with mutant METTL14-lentivirus (e), wild type or mutant METTL3-lentivirus (f), shMETTL14-

(g) or shMETTL3- (h) lentivirus or FTO-expressing lentivirus (i) as shown. (j) CLIP assays displaying the binding abundance between HNRNPA2B1 protein and m^6A site in HOKs against control IgG or anti-HNRNPA2B1 antibodies. (k) HNRNPA2B1 protein expression in HOKs with or without shHNRNPA2B1-lentivirus. n = 3. $^{**}P < 0.01$, $^{***}P < 0.001$ vs corresponding control; Ctrl, control. Data were expressed as means ± standard deviation. All experiments were carried out at least 3 times. Student's *t* test (b, d, e, g, h, i, j and k) and One-way ANOVA (f) were used for statistical analysis.

Uncropped blots (Supplementary Figure 7)

Figure 1b

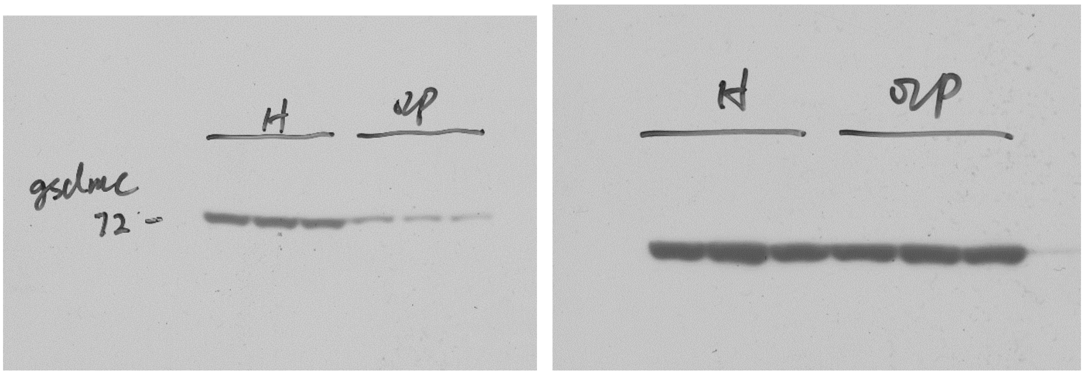

Figure 1e

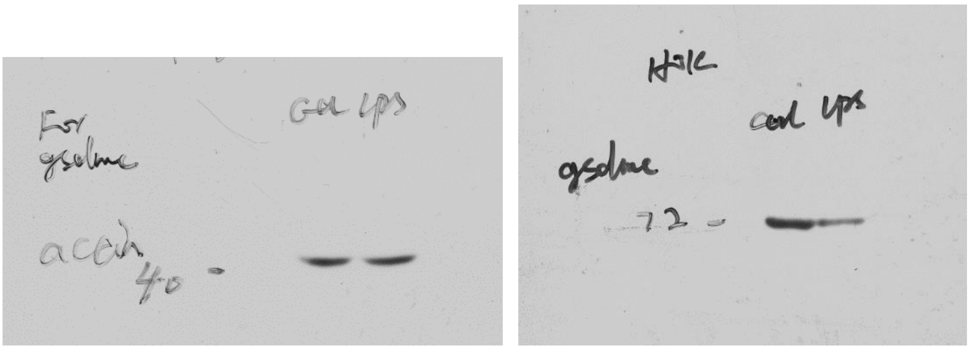

Figure 1h

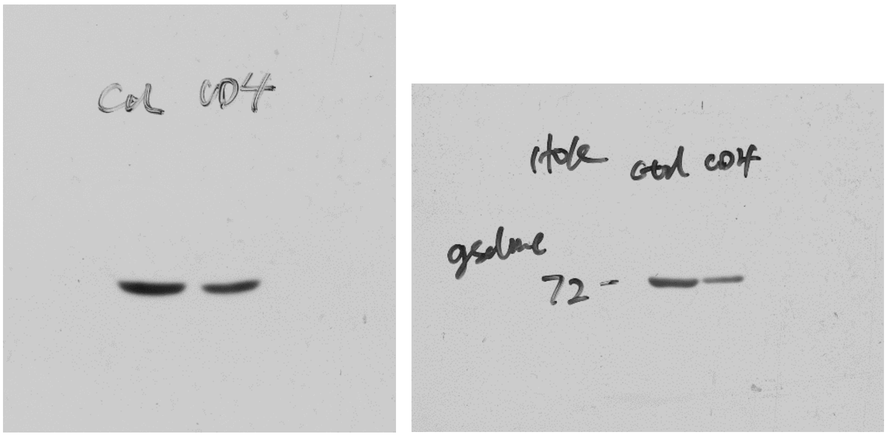

Figure 2f

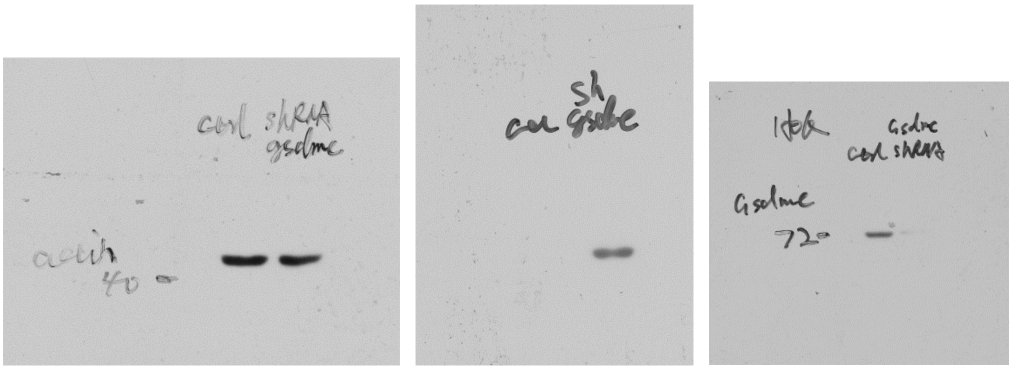

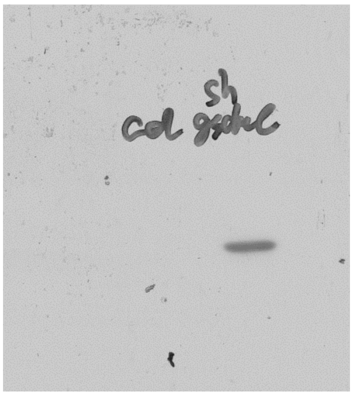

Figure 3d

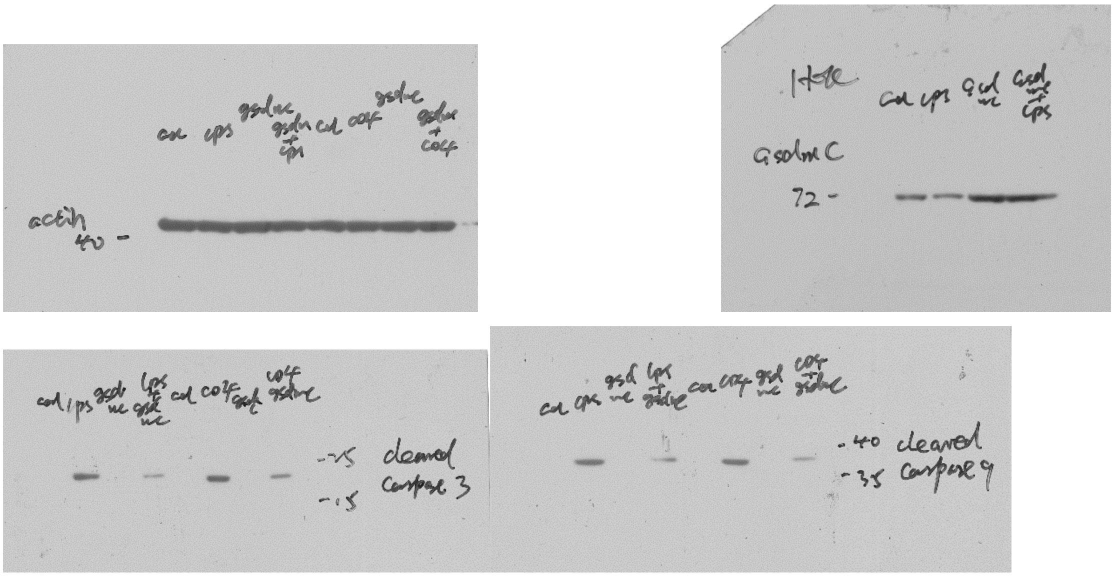

Figure 3h

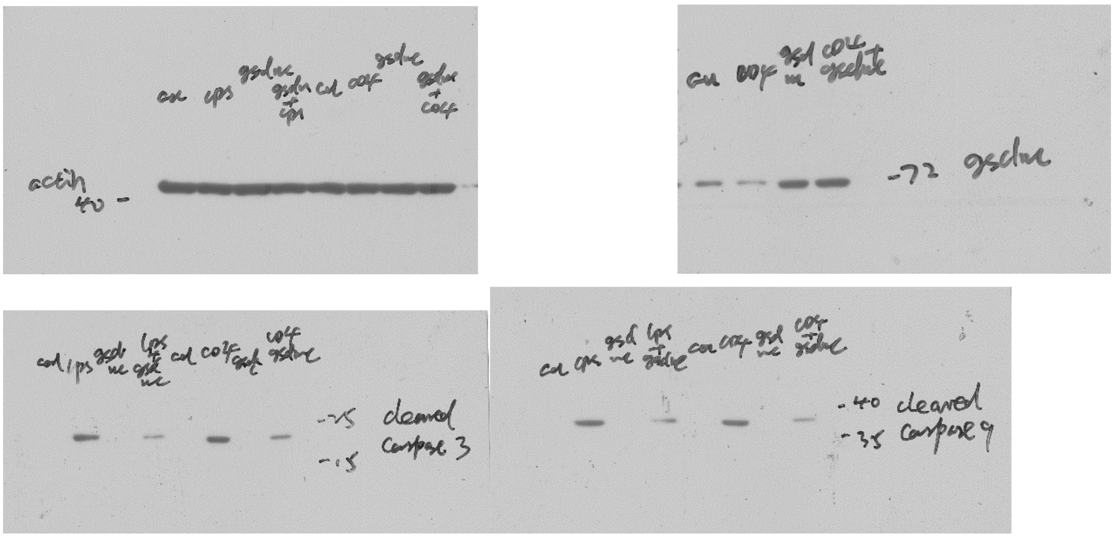

Figure 4c

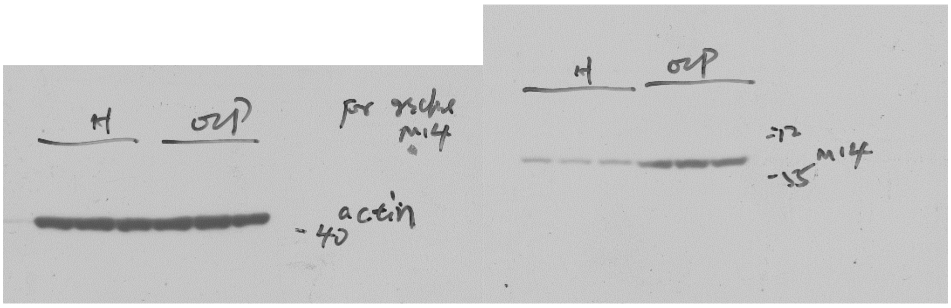

Figure 4e

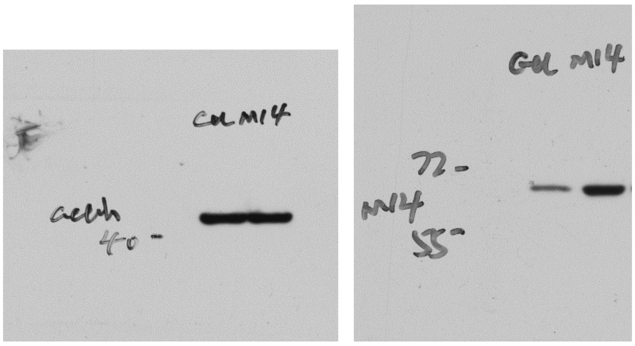

Figure 4j

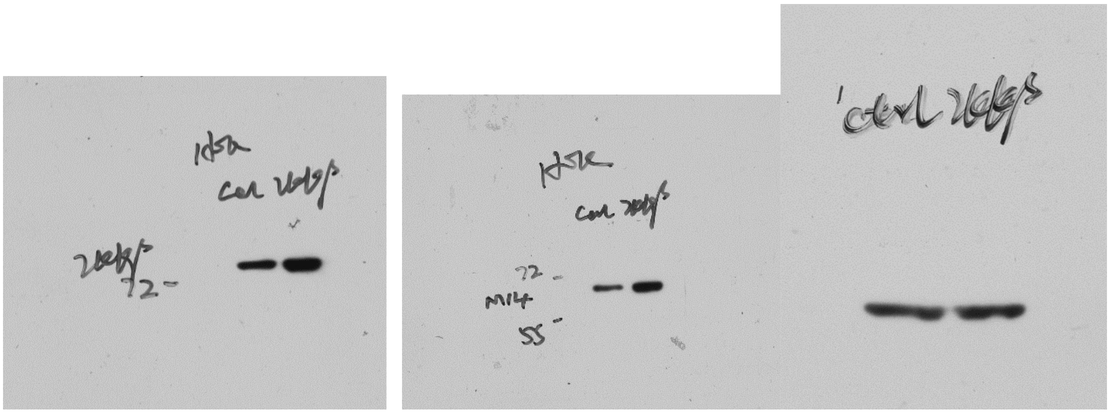

Figure 5e

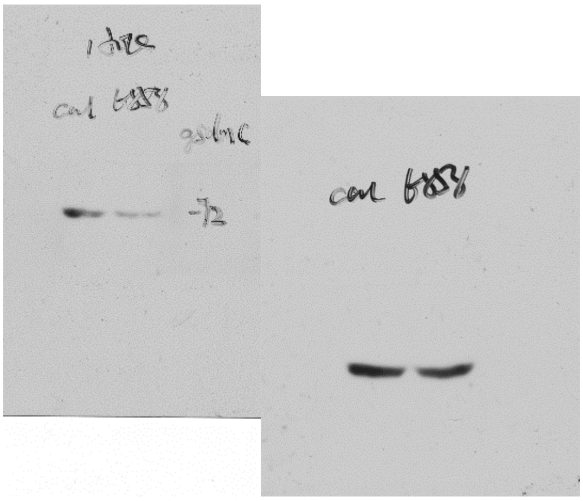

Figure 5g

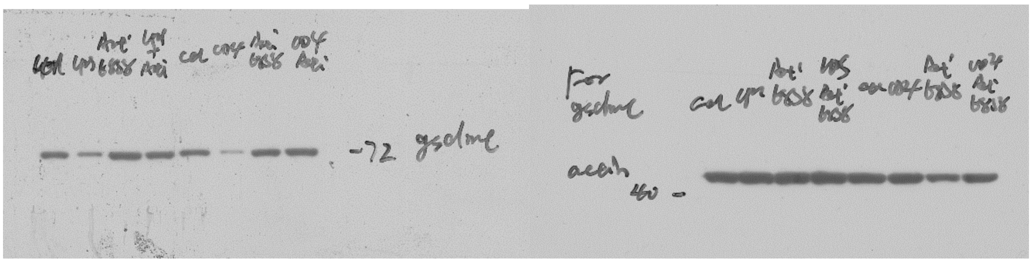

Figure 5i

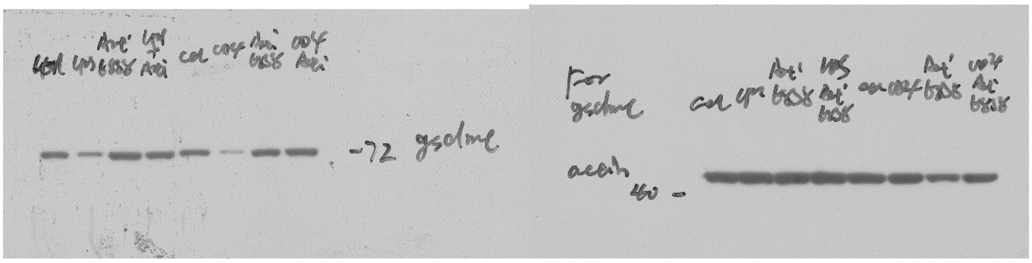

Figure 7 a-b

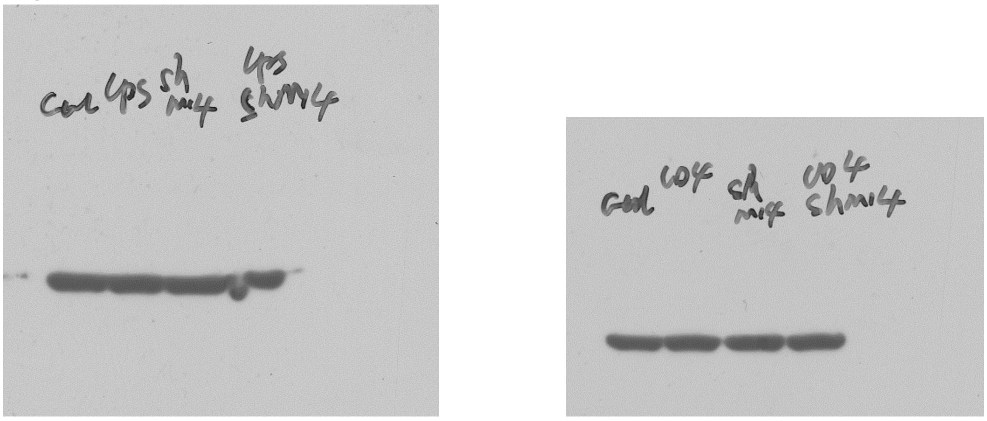

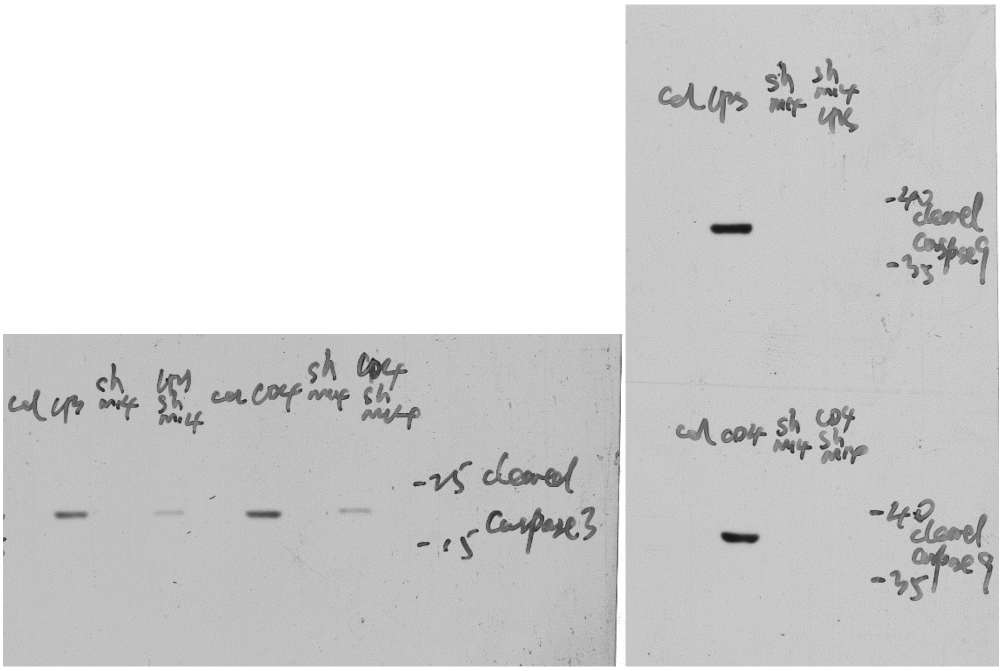

Figure 7 c-d

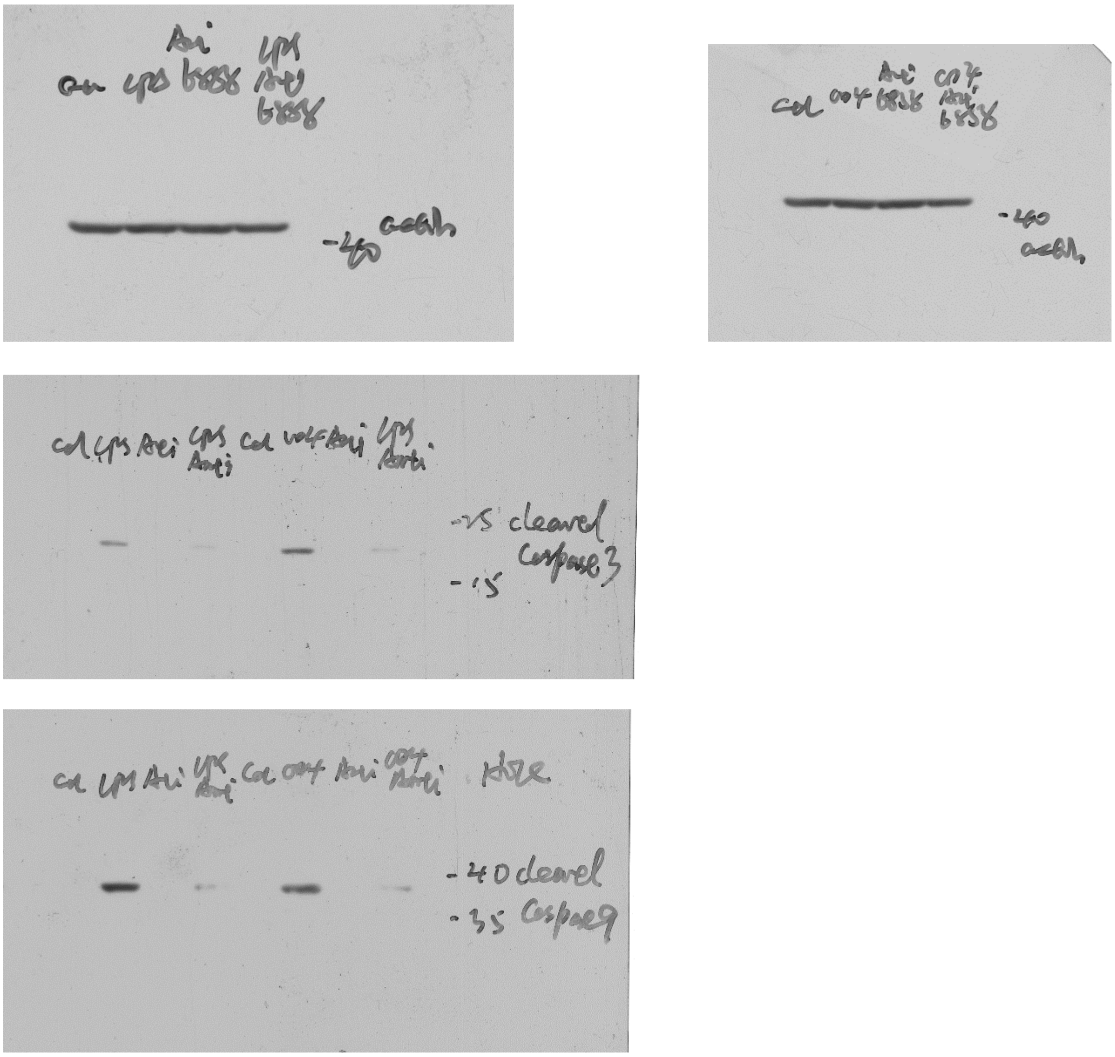

Figure 8e

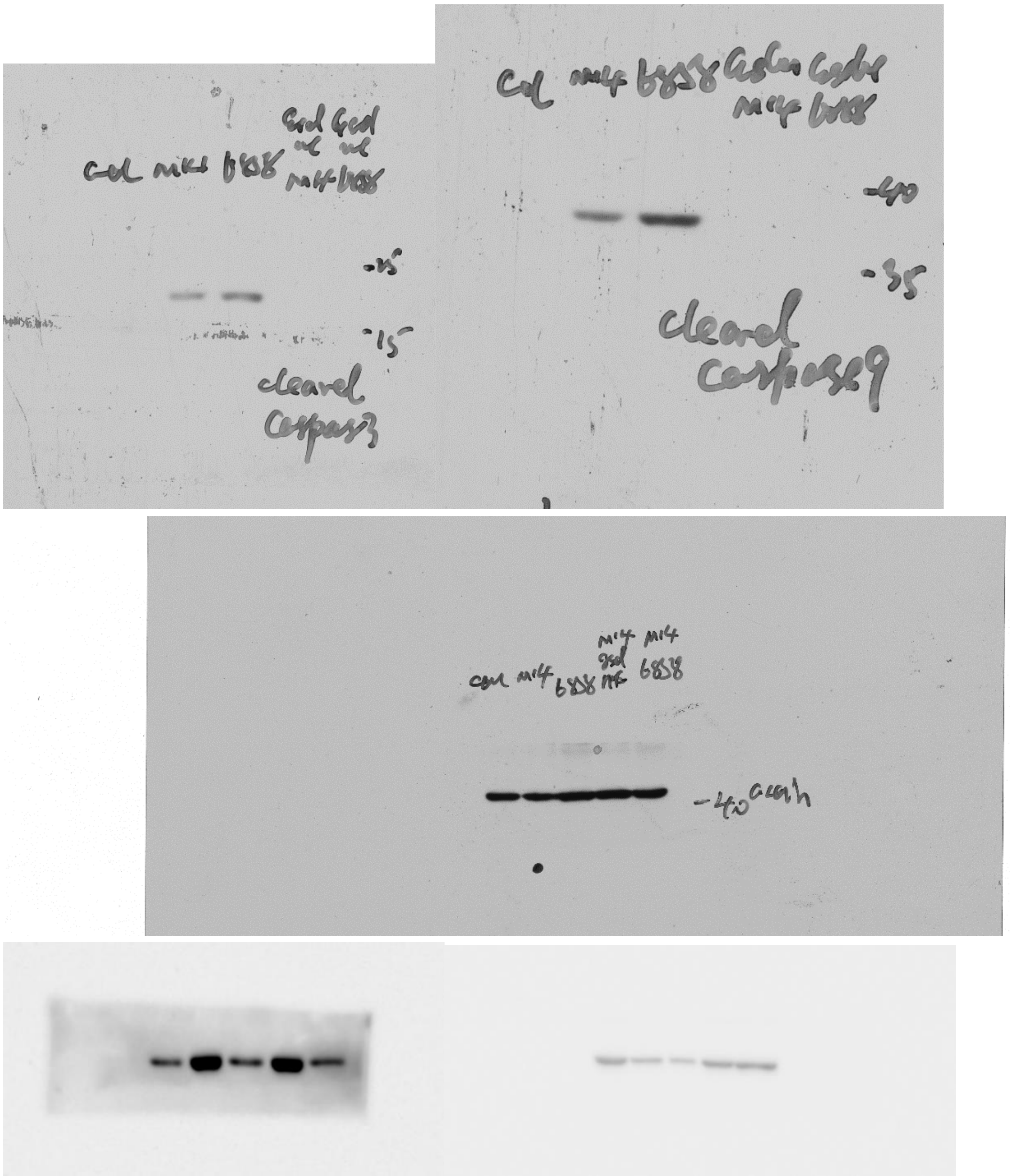

sup figure 1b

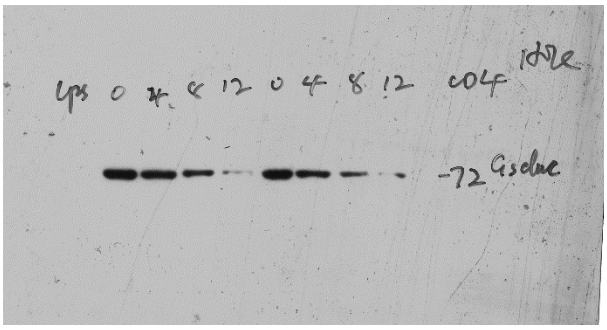

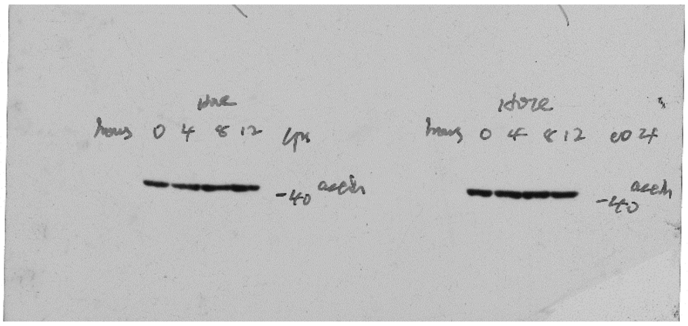

sup figure 2a

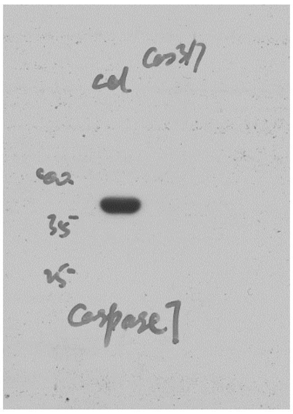

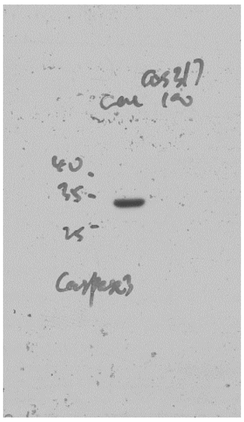

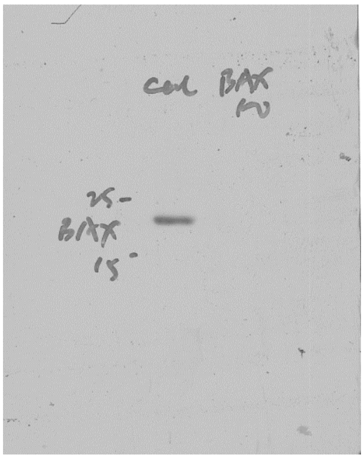

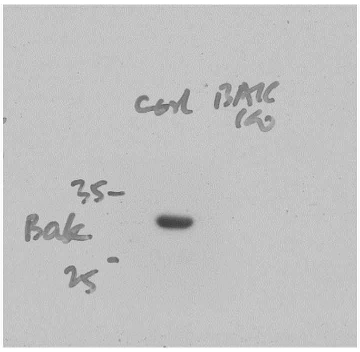

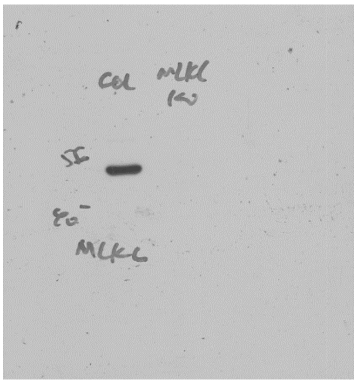

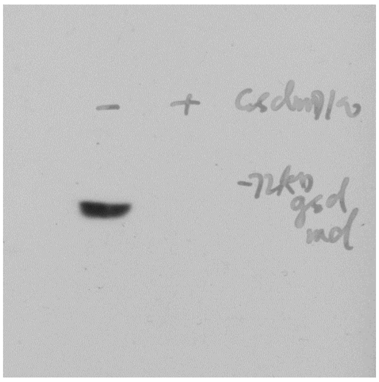

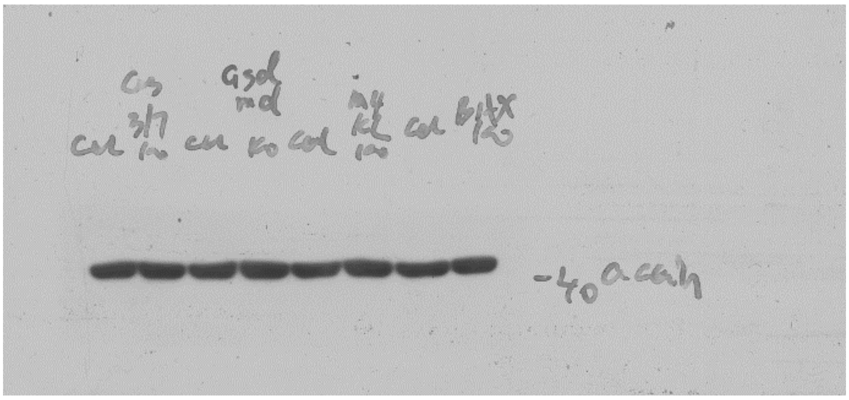

sup figure 4 i-j

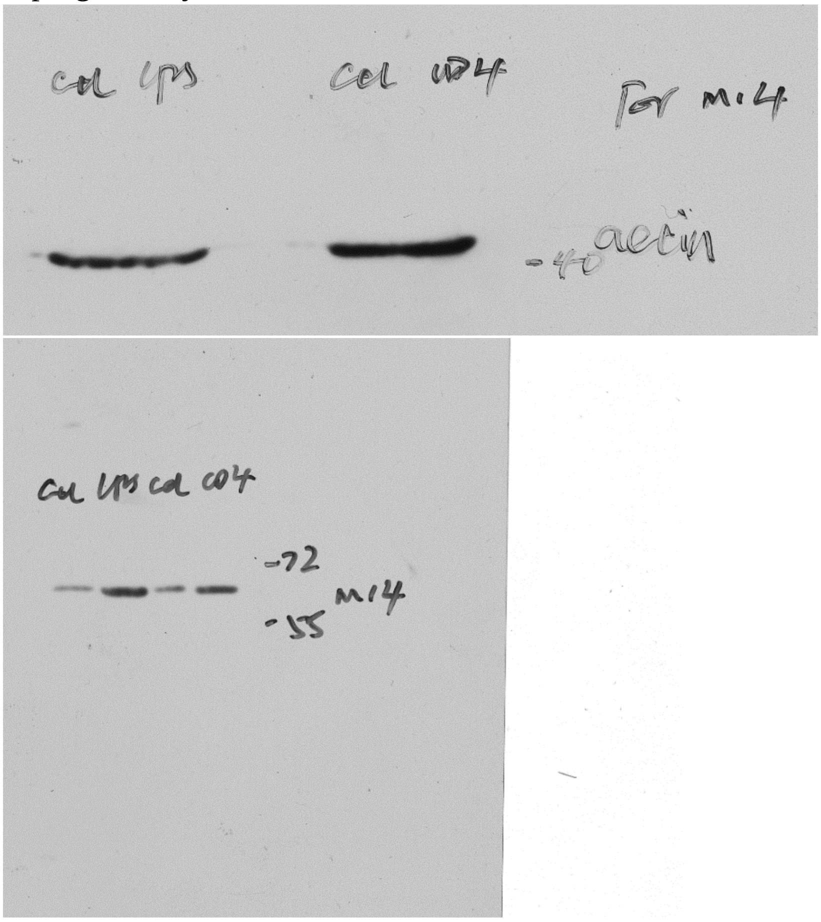

sup figure 6d

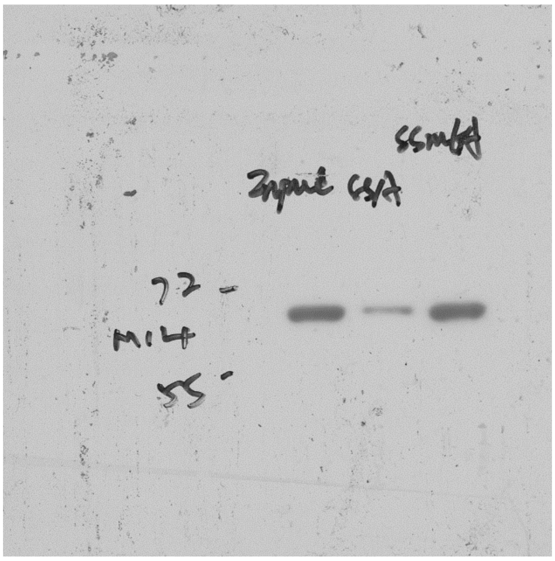

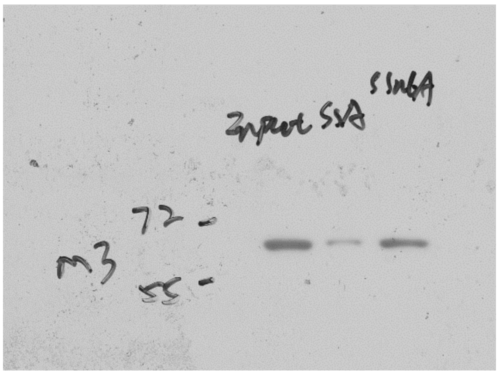

sup figure 6e

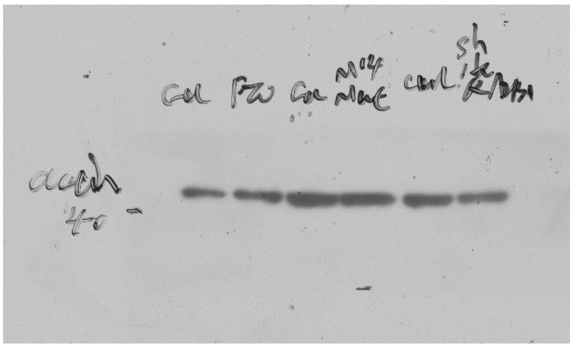

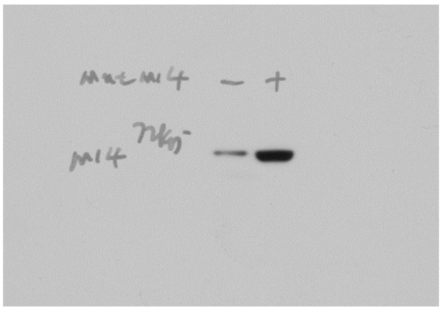

sup figure 6f

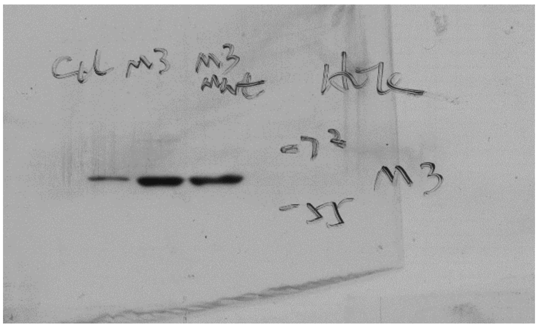

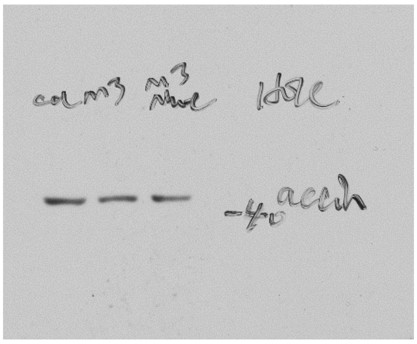

sup figure 6 g-h

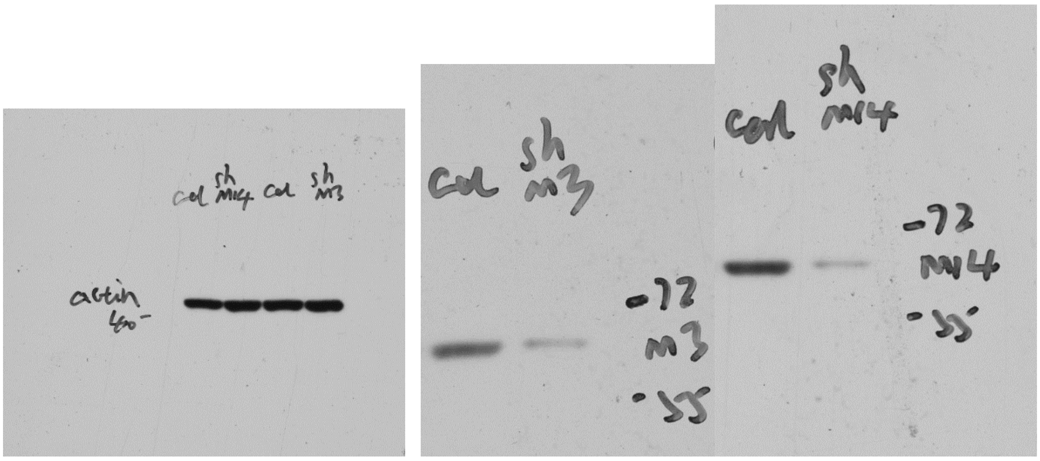

sup figure 6 i-k

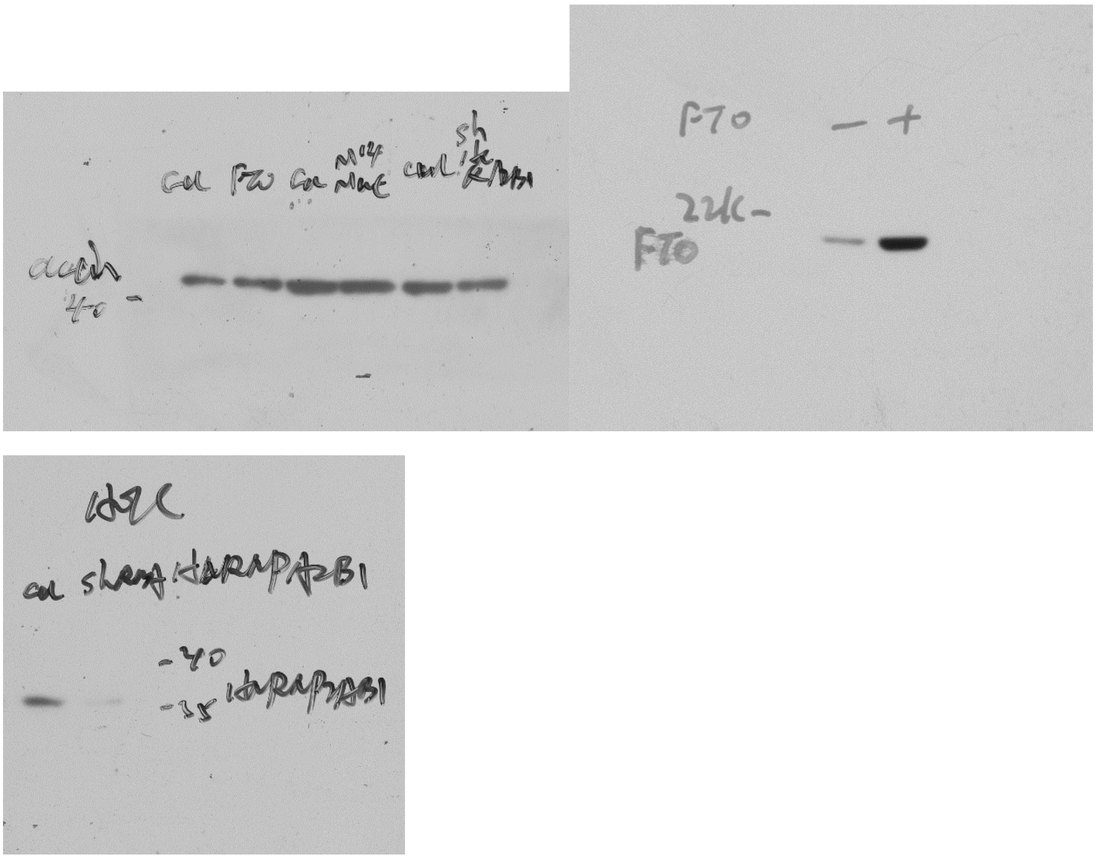

Supplementary tables

Supplementary Table 1

Antibodies information

Antibody	Source	Identifier
Anti-cleaved caspase 3	Cell Signaling Technology	Cat#: 9664S
Anti-cleaved caspase 9	Cell Signaling Technology	Cat#: 9505
Anti-p65	Cell Signaling Technology	Cat#: 3034
Anti-IKKβ	Cell Signaling Technology	Cat#: 8943
Anti-Caspase3	Cell Signaling Technology	Cat#: 9662
Anti-Caspase7	Cell Signaling Technology	Cat#: 9492
Anti-MLKL	Cell Signaling Technology	Cat#: 14993
Anti-METTL3	Aviva Systems Biology	Cat#: ARP39390_T100
Anti-HNRNPA2B1	Abcam	Cat#: ab31645
Anti-FTO	Abcam	Cat#: ab92821
Anti-m^6A	Synaptic Systems	Cat#: 202003

Anti-β-actin	Santa Cruz Biotechnology	Cat#: sc-47778
Anti-METTL14	Sigma-Aldrich	Cat#: HPA038002
Anti-GSDMC	ProteinTech	Cat#: 27630-1-AP
Anti-GSDMD	ProteinTech	Cat#: 20770-1-AP

Supplementary Table 2

Primers involved in this study.

Primer name	Forward(5′-3′)	Reverse(5′-3′)
METTL14 mutant	AACTGTGAAGCCTAGCACAGACG	CCTTTGATCCCCATGAGG
METTL3 mutant-1	AGCTTCTAGAGCCACCATGTACCCATACGATGTTCCAGATTACGCTTCGGACACGTGGAGCTCTAT	ATATCCGCGGGTGGGGCAGCCATCACAACTGCAAA
METTL3 mutant-2	GCCCCACCCGCGGATATTCACATGGAACTGCCCTAT	AATTGCGGCCGCCTATAAATTCTTAGGTTTAG
METTL14 plasmids	ATGGATAGCCGCTTGCAGGAGATCCGG	AATAGCTCCACCTTTCGGTGGAGA
METTL3 plasmids	ATGTCGGACACGTGGAGCTCTATCCA	GATATTTAAGAATCCAAATCTCTACTATG
FTO plasmids	ATGAAGCGCACCCCGACTGCCGAGGAAC	GATCCCAAAACGAAGGTCTTCGACTGGAGA
GSDMC plasmids(FL)	ATGCCCTCCATGTTGGAACGCATTAGC	AATCCGGAGTCGGTCGACGACGTCG
GSDMC	TCCATGTTGGAACGCATTAGC	CAAACTGACGTAATTTGGTGGC

METTL14	AGTGCCGACAGCATTGGTG	GGAGCAGAGGTATCATAGGAAGC
METTL3	TTGTCTCCAACCTTCCGTAGT	CCAGATCAGAGAGGTGGTGTAG
WTAP	CTTCCCAAGAAGGTTCGATTGA	TCAGACTCTCTTAGGCCAGTTAC
FTO	ACTTGGCTCCCTTATCTGACC	TGTGCAGTGTGAGAAAGGCTT
ALKBH5	CGGCGAAGGCTACACTTACG	CCACCAGCTTTTGGATCACCA
GAPDH	ACCACAGTCCATGCCATCAC	TCCACCACCCTGTTGCTGTA
hsa-miR-6858	GTGAGGAGGGGCTGGCACCCAC	
hsa-primiR-6858	CCAGCCGCTGGGAACCCTACGACC	ACACTCCTGTTCCACCCGTCTCCTC
hsa-miR-4795-3p	ATATTATTAGCCACTTCTGGAT	
hsa-miR-548ax	AGAAGTAATTGCGGTTTTGCCA	
hsa-miR-548ao	AGAAGTAACTACGGTTTTTGCA	
hsa-miR-4689	TTGAGGAGACATGGTGGGGGCC	
U6	GATGACACGCAAATTCGTGAA	
m^6A RIP	CAAGGTGGTGCTGAGGAGCT	GCACATGAACAGGGTGAAGAAAA

and CLIP	GGTA	GTCCC
ChIP	GGACTGTCATCTGTGGAATCA ATTGA	CAGACTTCCTCCTGTCAGTCGACT GAAGAGG
Human GSDMC-m^6A site 1	ACTGTTGTGACAGGACCGTT	ACTCCTTCAGAAATGATGGC
Human GSDMC-m^6A site 2	TGACAGAGGCTGTTGAACTG	CTCCTTGATAACCAGCTGCT
Human GSDMC-m^6A site 3	AAGGATGTTCAGGATGTCAT	TTAAACCATGCATGGTTTGA
Human GSDMC-m^6A site 4	AAGAGATAGGGCTGTGCCTC	CTCTTGTCAATATATAGAGT
