# Supplementary material for: METTL14-upregulated miR-6858 triggers cell apoptosis in keratinocytes of oral lichen planus through decreasing GSDMC
Source: Commun Biol. 2023 Sep 23;6:976. doi: 10.1038/s42003-023-05360-6 (PMC10517968; doi:10.1038/s42003-023-05360-6)
Supplement: Supplementary file 3 — Description of Additional Supplementary Files [file 42003_2023_5360_MOESM3_ESM.pdf]

Description of Additional Supplementary Files

File name: Supplementary Data 1
Description: The source data concerning caspase activity assessment behind the graphs in the paper.

File name: Supplementary Data 2
Description: The source data concerning CLIP, ChIP and luciferase report assays behind the graphs in the paper.

File name: Supplementary Data 3
Description: The source data concerning m6a detections behind the graphs in the paper.

File name: Supplementary Data 4
Description: The source data concerning PCR assays behind the graphs in the paper.

File name: Supplementary Data 5
Description: The source data concerning western blot assays behind the graphs in the paper.
